# A proposed treatment scheme for chronic recurrent multifocal osteomyelitis (CRMO): a case series of nine patients

**DOI:** 10.1186/1546-0096-12-S1-P239

**Published:** 2014-09-17

**Authors:** Ezgi Deniz Batu, Bora Gulhan, Rezan Topaloglu, Seza Ozen

**Affiliations:** 1 Pediatric Rheumatology, Hacettepe University, Ankara, Turkey; 2 Pediatric Nephrology, Hacettepe University, Ankara, Turkey

## Introduction

CRMO is a rare, orphan auto-inflammatory disease characterized by recurrent episodes of pain associated with sterile bone inflammation. There is no consensus on the optimal treatment.

## Objectives

The aim of this study was to review our CRMO patients and report our results with etanercept treatment in patients with a resistant course in CRMO.

## Methods

Retrospective descriptive case series of nine children diagnosed as CRMO at the Department of Pediatric Rheumatology at Hacettepe University, Ankara, Turkey. Disease activity was assessed by acute phase reactants (ESR, CRP), physician and patient/parent visual analogue scales (VAS) (0-10 cm), and radiological findings (MRI and bone scintigraphy).

## Results

The median age of symptom onset was 8,2 years, while the patients were diagnosed to have CRMO at a median of 10 years of age. Eight out of nine patients presented with extremity pain, while five of the patients had back pain on presentation. Eight out of nine patients presented with extremity pain, while five had back pain on presentation. The patients had a median of three lesions at the time of diagnosis. There was sacroiliitis in seven patients. Four out of nine patients were treated with nonsteroidal anti-inflammatory drugs (NSAID) and methotrexate (MTX). Five patients did not respond to these treatments, thus etanercept therapy was started at an initial dose of 0,8 mg/kg/week after a median of 24 months from diagnosis. Acute phase reactants returned to normal, physician and patient/parent visual analogue scales significantly improved in all patients after etanercept treatment. We were able to show healing of lesions in imaging that correlated with the improvement of VAS and pain in the patients, suggesting that anti-TNF was effective on the bone inflammation. We have extended the time interval between two doses of etanercept from one week to two weeks after six months of remission with maintenance of the complete response in four of these patients.

## Conclusion

Our treatment policy in CRMO is to start with NSAIDs and MTX. If the patient is refractory to these drugs, anti-TNF treatment is commenced. Anti-TNF treatment was effective in the treatment of our CRMO patients, We suggest that extending the dosing interval is an effective option once they are in remission for 6 months. We also suggest that imaging may be included as an outcome measure in patients with CRMO. Further prospective studies are needed to determine the optimum outcome measures and treatment strategy.

## Disclosure of interest

None declared.

